# Perioperative local anesthetic and antibiotic considerations during Mohs micrographic surgery on a patient with myasthenia gravis

**DOI:** 10.1016/j.jdcr.2026.09.011

**Published:** 2026-09-12

**Authors:** Jin Nathan Swun, Ryan Dieringer, Adam M. Rotunda

**Affiliations:** a Cape Fear Valley Health School of Medicine, Methodist University, Fayetteville, North Carolina; b Samueli School of Engineering, University of California, Los Angeles, California; c Department of Dermatology, University of California, Irvine, California; d Private Practice, Newport Skin Cancer, Newport Beach, California

**Keywords:** anesthesia, antibiotic considerations, bupivacaine, cephalexin, cephalosporin, lidocaine, local anesthetic, Mohs micrographic surgery, myasthenia gravis, patient, perioperative

## Introduction

Patients with myasthenia gravis (MG) are susceptible to medication-induced exacerbations from anesthetics and antibiotics used in Mohs micrographic surgery (MMS) that may inadvertently disrupt neuromuscular signaling.^1^^,^^2^ These exacerbations include increased muscular weakness, bulbar symptoms, and respiratory compromise, which may escalate to an MG crisis, requiring intubation.^1^^,^^2^

Here, we review perioperative medication considerations for patients with MG undergoing MMS, with a focus on local anesthetic selection and antibiotic classes that may worsen neuromuscular weakness or precipitate myasthenic crisis in susceptible patients.

## Case report

A 67-year-old man underwent MMS for a 0.7 × 0.7 cm nodular basal cell carcinoma on the right medial cheek. On examination, the patient had mild right eyelid ptosis, the sole manifestation of untreated MG diagnosed 2 weeks prior to surgery (Figs 1 and 2). Our patient’s neurologist advised against lidocaine, recommending bupivacaine as an alternative, and cephalexin if antibiotics were required. These suggestions guided our care for this patient.

**Fig 1:**
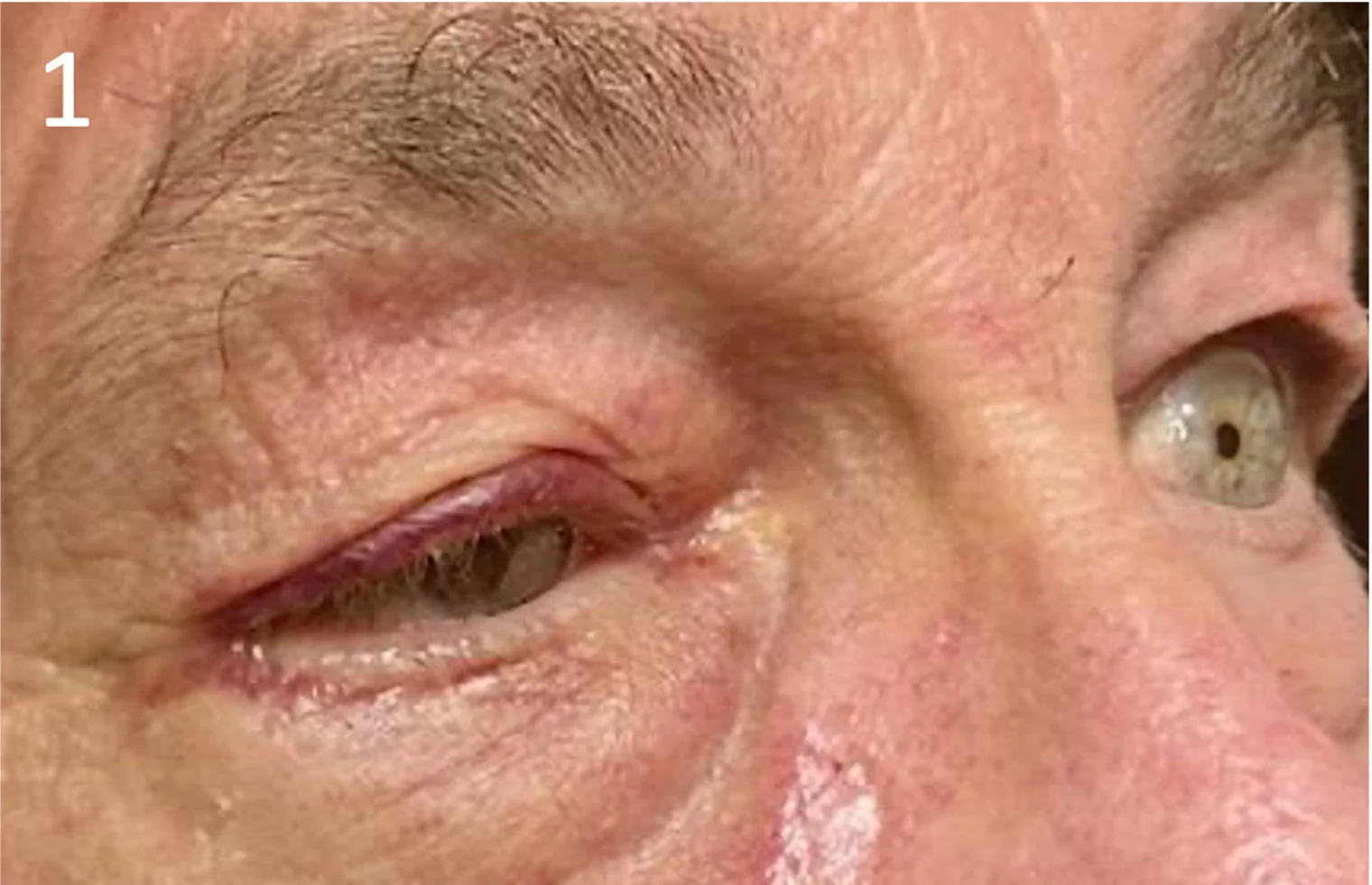
Right lid ptosis at 1 week following MMS and reconstruction on the right medial cheek, with mild ipsilateral infraorbital swelling and ecchymoses. Ptosis was noted preoperatively, though baseline photographs did not capture eyelid position.

**Fig 2:**
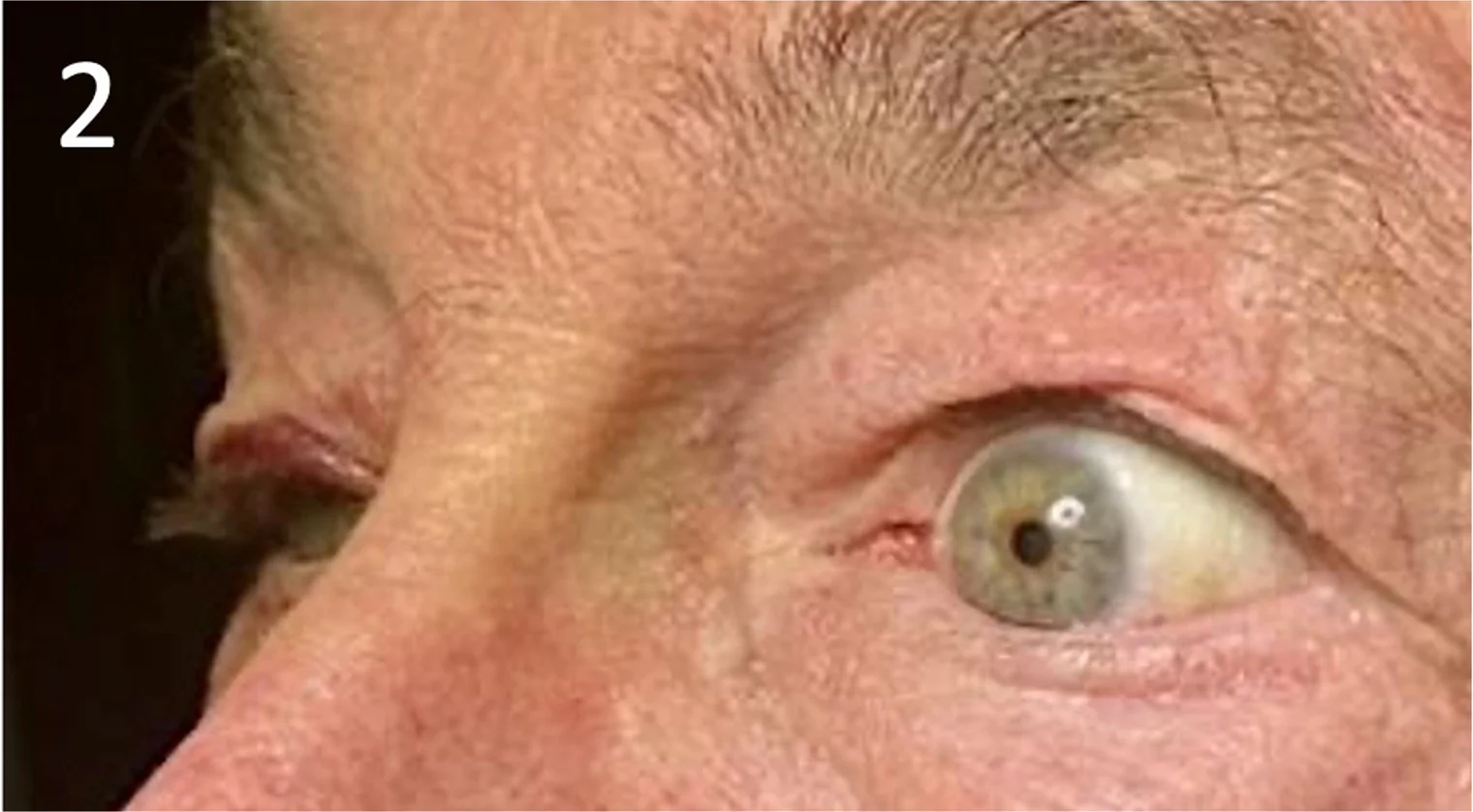
Contralateral side without lid ptosis, unchanged from the preoperative photograph, not shown.

While the patient’s neurologist did not specify a maximum dose of bupivacaine, local administration was limited to the dose necessary to sustain adequate anesthesia during MMS, within the recommended adult maximum dose of 175 mg for local infiltration.^3^ The patient was anesthetized with approximately 9 mL of 0.25% (22.5 mg total) bupivacaine hydrochloride (Pfizer) without epinephrine, divided evenly prior to his first stage and after his second and last Mohs stage. The resulting 1.5 × 0.8 cm defect was closed in a linear fashion with interrupted 5-0 Monocryl (poliglecaprone 25, Ethicon, Johnson and Johnson) and running 5-0 Prolene (polypropylene, Ethicon, Johnson and Johnson) sutures. Aside from moderate intraoperative bleeding, the patient tolerated the procedure well without new or worsening myasthenic symptoms.

After discussing the risks and benefits of prophylactic antibiotics, the patient elected to take cephalexin as a precaution, given the degree of intraoperative bleeding and potential risk for wound infection. At 1-week suture removal, the patient reported no adverse medication reactions and noted mild, unexpected improvement in his right eyelid ptosis (Figs 1 and 2).

Although perioperative medication for MG patients is discussed in other surgical settings, their selection for MMS patients has not been specifically described.^1^^,^^2^

## Discussion

### Preoperative considerations

Understanding the pathophysiology of MG guided our management of this MMS patient, including the use of 0.25% bupivacaine and cephalexin. MG is an autoimmune disorder of neuromuscular transmission in which impaired acetylcholine (ACh) signaling at the neuromuscular junction (NMJ) produces fluctuating skeletal muscle weakness, with potential ocular, bulbar, limb, and respiratory involvement.^4^ In most patients, pathogenic autoantibodies target postsynaptic acetylcholine receptors (AChR), leading to receptor blockade or destruction and consequent failure of neuromuscular signaling.^4^ Before the procedure, the Mohs surgeon performed a physical examination, noting no additional myasthenic symptoms other than right eyelid ptosis.

While patients with MG may be regularly taking acetylcholinesterase inhibitor medication as a standard treatment or selectively undergoing intravenous immunoglobulin (IVIg) therapy, they should avoid agents, such as certain neuromuscular blocking agents and antibiotics which may exacerbate preexisting or new symptoms during surgery (Table I).^2^^,^^5^ These considerations were important to review prior to MMS, and why coordinating care with the patient’s neurologist is essential.

**Table I:** Antibiotic classes that are considered potentially harmful or relatively safe for the perioperative care of MG patients undergoing MMS, based upon current literature^1^^,^^2^

Class (examples)	Associated with MG exacerbations (mechanism)
Fluoroquinolone (ciprofloxacin, levofloxacin)	Yes, latency period ranging from 15 min to 5 d, with increased MG exacerbations (quinolone moiety shown to physically block the AChR channel)
Macrolide (azithromycin, erythromycin)	Yes, latency period ranging from minutes to days, with generalized MG exacerbations (reduced receptor endplate sensitivity to ACh)
Aminoglycoside (gentamicin, tobramycin)	Yes, high doses cause rapid, severe exacerbations (reduced presynaptic ACh release and postsynaptic blocking of ACh)
Penicillin (amoxicillin, dicloxacillin)	Yes (limited evidence), rare, reported MG exacerbations occurring days after exposure to amoxicillin (mechanism unclear)
Cephalosporin (cephalexin, cefdinir)	N/A
Tetracycline (minocycline, doxycycline)	N/A
Sulfonamide (sulfamethoxazole, TMP-SMX)	N/A

*ACh*, Acetylcholine; *AChR*, acetylcholine receptor; *MG*, myasthenia gravis; *MMS*, Mohs micrographic surgery; *TMP-SMX*, trimethoprim/sulfamethoxazole.

### Intraoperative considerations

MMS may be valuable for appropriately selected patients with MG as it is performed under local anesthesia, avoiding general anesthesia and neuromuscular blocking agents. Careful selection and dosing of local anesthetic was key during this procedure as the concern with local anesthetics on a patient with MG is related to cumulative dose and elevated systemic exposure.^2^ The patient was carefully monitored after injections with bupivacaine to assess cutaneous discomfort and exacerbation or new onset of MG symptoms.

Although both lidocaine and bupivacaine are amide local anesthetics, bupivacaine was selected for its longer duration of action rather than clear evidence that bupivacaine is intrinsically less likely to exacerbate MG. Lidocaine 1% with epinephrine is the conventional local anesthetic used in cutaneous surgery. It has a more rapid (within seconds) onset, but is a shorter-acting anesthetic (1-2 hours without epinephrine vs 2-6 hours with epinephrine) relative to bupivacaine, which has a slower onset (<5 minutes) but is a longer-acting anesthetic (7-9 hours with or without epinephrine).^6^^,^^7^

In our patient, bupivacaine without epinephrine provided sustained anesthesia throughout both MMS and reconstruction (spanning several hours), reducing reinjections between stages, and limiting cumulative anesthetic exposure. This approach was also chosen to minimize the theoretical risk of exacerbating eyelid weakness, given the proximity of the basal cell carcinoma nodule to the right eye. Because bupivacaine with epinephrine was unavailable at the time of surgery, the absence of vasoconstriction likely contributed to the sustained intraoperative bleeding.

### Postoperative considerations

The selection of prophylactic antibiotics was key in the perioperative management of the patient’s MG. Our patient was prescribed cephalexin, a first-generation cephalosporin, due to concerns of other classes of antibiotics triggering MG symptoms. Other classes of antibiotics, such as fluoroquinolones, macrolides, and aminoglycosides, have been associated with exacerbating myasthenic symptoms, as they interrupt pre- or postsynaptic ACh activity at the NMJ (Table I).^1^^,^^2^ However, current literature has not associated cephalosporins with MG exacerbation, which supported our rationale for selecting cephalexin as our choice of prophylactic antibiotics for our patient.

Postoperative care for our patient also involved monitoring for new or worsening myasthenic symptoms, assessing pain or discomfort immediately after the procedure and days after MMS. Immediate neurology follow-up should be considered if MG symptoms worsen.

The patient was also advised to take two 325 mg acetaminophen tablets with two 200 mg nonsteroidal anti-inflammatory drug (NSAID) tablets as needed for postoperative pain within a 24-hour period. The patient elected not to and reported no significant postoperative pain.

While no complications associated with MG arose during MMS or were reported by the patient postoperatively, our perioperative management for this patient served as a preventive care strategy to minimize the risk of medication-induced MG exacerbation. Preoperative, intraoperative, and postoperative precautions were taken to ensure the patient’s safety during the course of MMS.

### Declaration of generative AI and AI-assisted technologies in the writing process

During the preparation of this work, the authors used ChatGPT in order to improve syntax and grammar. After using this tool/service, the authors reviewed and edited the content as needed and take full responsibility for the content of the publication.

## Conflicts of interest

None disclosed.
